# Broadband unidirectional twin-element MIMO antenna scheme for mid-band 5G and WLAN laptops

**DOI:** 10.1038/s41598-024-60346-6

**Published:** 2024-04-27

**Authors:** Bancha Luadang, Pisit Janpangngern, Khanet Pookkapund, Sitthichai Dentri, Monai Krairiksh, Chuwong Phongcharoenpanich

**Affiliations:** 1 Faculty of Engineering, Rajamangala University of Technology Rattanakosin, Nakhon Pathom, 73170 Thailand; 2 School of Engineering, King Mongkut’s Institute of Technology Ladkrabang, Bangkok, 10520 Thailand; 3 Faculty of Science and Technology, Phranakhon Si Ayutthaya Rajabhat University, Phranakhon Si Ayutthaya, 13000 Thailand; 4 College of Industrial Technology, King Mongkut’s University of Technology North Bangkok, Bangkok, 10800 Thailand

**Keywords:** Broadband antenna, Coupling gap, Integrated antenna, MIMO antenna, 5G antenna, Electrical and electronic engineering, Physics

## Abstract

This research proposes a broadband unidirectional twin-element multiple-input-multiple-output (MIMO) antenna scheme for mid-band 5G and WLAN applications. The twin-element antenna scheme comprises two single-element antennas, and each single-element antenna consists of a T-shaped hemispherical feeding patch, left- and right-arm radiating patches, and a conjoined triangular ground plane. The twin-element MIMO antenna scheme is integrated with a laptop model functioning as the reflector. The measured impedance bandwidth (|*S*_11_|, |*S*_22_|≤ − 6 dB) are 55.32%, covering 3.4–6.0 GHz, and the measured mutual coupling (|*S*_12_|) is less than − 15 dB. The measured gain at the center frequency (4.5 GHz) is 4.585 dBi. Besides, the measured xz- and yz-plane cross-polarization levels are below − 25 dB and − 15 dB, respectively. The half-power beamwidth (HPBW) in the xz-plane at 3.5, 4.5, and 5.5 GHz are 99°, 92.8°, and 84.2°, and the corresponding HPBW in the yz-plane are 102°, 78°, and 102°. The measured xz- and yz-plane back lobe levels are below − 15 dB across the entire operating frequency band (3.5–5.5 GHz). The radiation pattern of the twin-element MIMO antenna scheme is of unidirectionality. Furthermore, the envelope correlation coefficient and diversity gain of the twin-element antenna scheme are < 0.001 and > 9.99 dB, respectively. The proposed broadband unidirectional twin-element MIMO antenna scheme is thus operationally suitable for mid-band 5G/WLAN communication systems. Essentially, this research is the first to propose a broadband twin-element MIMO antenna scheme for mid-band 5G/WLAN applications.

## Introduction

The fifth generation (5G) of wireless cellular technology offers faster data rates, lower latency, more reliable connections and capacity than previous cellular generations^1–3^. As a result, the 5G technology is ideal for applications that require fast and real-time data transmission^4–7^.

MIMO (multiple input, multiple output) is an antenna technology for wireless communications in which multiple antennas are used at both the source (transmitter) and the destination (receiver). MIMO antennas achieve faster data speed and higher transmission capacity as data can travel over many signal paths at the same time. The MIMO antennas can overcome interference, multipath fading, and radiation losses. The MIMO technology is thus essential for 5G applications to achieve low latency, maximum throughput, and high efficiency.

Different MIMO antennas for 5G mobile terminals were proposed to improve impedance bandwidth, reduce mutual coupling, and enhance isolation. In^8^, a three-slot printed wideband antenna operable in GSM and LTE bands achieved high antenna efficiency, bandwidth, and channel capacity. In^9^, an eight-element antenna with neutralization lines and ground slots also achieved high efficiency, bandwidth, channel capacity, and compact size.

In^10^, a self-adaptive MIMO antenna could achieve good decoupling and impedance matching, and a technique for decoupling inverted-F antennas (IFAs) by utilizing high-order ground plane modes has been proposed for a 5G mobile MIMO platform operating at 3.5 GHz^11^. In^12^, an eight-element uniplanar MIMO antenna with polarization diversity could reduce mutual coupling and improve interference and fading resistance. Besides, a four-element multi-band antenna with the common ground plane was proposed in^13^; and a multi-element antenna with balanced slot mode and polarization diversity was presented in^14^.

To improve data throughput and radiation efficiency of MIMO antennas, monopolar patch antennas (MPA) with monopole radiation patterns were deployed as element antennas in the antenna schemes^15–20^. In^21^, a Y-type MPA could achieve an antenna efficiency of 88% and envelope correlation coefficient (*ECC*) of 0.1, rendering it suitable for 5G access points. A four-port MIMO antenna was used in underlay and interweave cognitive radio^22^, and a wide-band MIMO antenna with frequency tuning was proposed in^23^.

In addition, neutralization lines^24,25^ and decoupling techniques^26,27^ were employed to improve isolation. In^28^, a pair of compact self-decoupled antennas with two antenna elements on the common ground plane could achieve good isolation, low *ECC*, and high antenna efficiency. In^29–32^, polarization diversity and F-shaped stubs were used in MIMO antennas to achieve high isolation and low *ECC*. Specifically, a 16-element antenna achieved high gain, stable radiation pattern, enhanced isolation, and wide bandwidth^32^.

A four-port omnidirectional MIMO antenna was proposed in^33^. In^34^, a planar inverted-F antenna scheme, consisting of two antenna elements and four ports, was proposed for use in cellular applications and 5G IoT; and the isolation and *ECC* of the planar inverted-F antenna scheme were less than − 25 dB and 0.009. In^35^, a MIMO antenna with slots and rotating patches could achieve high isolation, and in^36^, a MIMO antenna with a T-shaped matching load, a decoupling dot-wall, and square slots aligned with the dot-wall also achieved high isolation.

A sub-6-GHz four-element MIMO slot antenna for 5G tablet computers was proposed in^37^; and the antenna was operable in the 3.4–3.6 GHz frequency band with high isolation and antenna efficiency. In^38^, the existing mobile terminal antennas were reviewed and their performance compared. In^39^, a two-element MIMO antenna was proposed for eight LTE bands. In^40^, a four-element MIMO antenna with short-circuit decoupling and decoupling chip inductors could achieve high isolation. In^41^, a dual-band MIMO antenna could achieve over 40% antenna efficiency.

However, existing 5G laptop MIMO antennas primarily support cellular frequencies (i.e., LTE and mid-band 5G). An ideal 5G/laptop MIMO antenna should be able to support both mobile and WLAN frequencies to enable reliable connectivity and data communication. Specifically, there exists no research on MIMO antennas that are capable of supporting both 5G and WLAN frequencies. This research is thus the first to propose a broadband twin-element MIMO antenna scheme for mid-band 5G/WLAN applications.

More specifically, this research proposes a broadband unidirectional twin-element MIMO antenna scheme for mid-band 5G and WLAN applications (3.5–5.5 GHz). The twin-element antenna scheme comprises two single-element antennas on FR4 substrate. The single-element antenna consists of a T-shaped hemispherical feeding patch, left- and right-arm radiating patches, and a conjoined triangular ground plane. The T-shaped hemispherical feeding patch is located between the left- and right-arm radiating patches separated by a coupling gap. The twin-element MIMO antenna scheme is integrated with a laptop model which functions as the reflector. Simulations and experiments are subsequently carried out and results compared. The performance metrics of the proposed twin-element MIMO antenna scheme include the impedance bandwidth, mutual coupling, antenna gain, radiation pattern, envelope correlation coefficient, and diversity gain.

## Antenna Structure

### Single Element Antenna Structure

**Figure 1:**
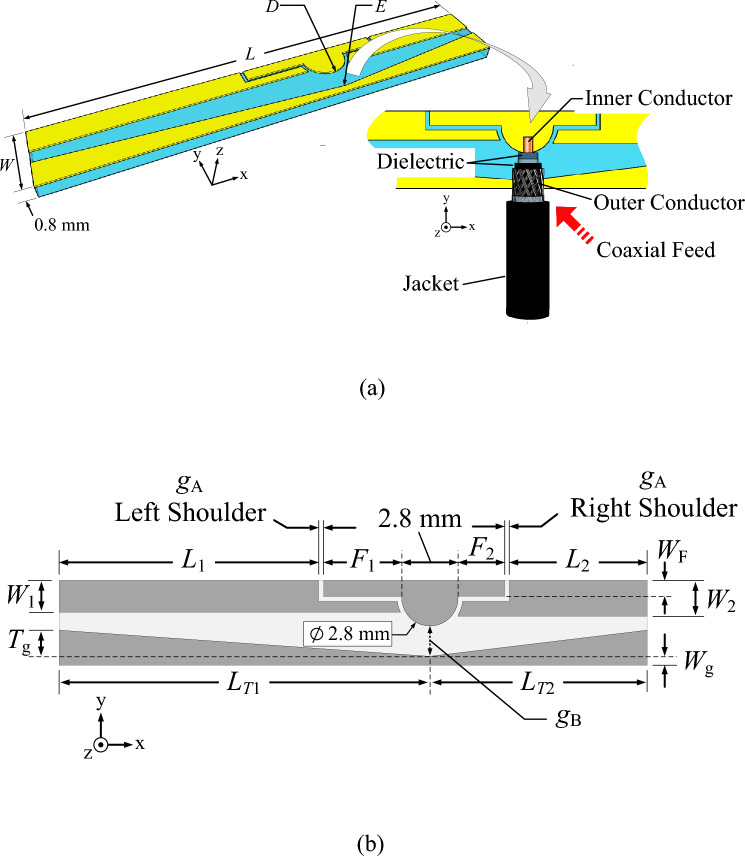
Geometry of the single element antenna (not to scale): (**a**) perspective view, (**b**) top view.

Figure 1 shows the geometry of the single-element antenna, consisting of three main components: a T-shaped hemispherical feeding patch, long- (left) and short-arm (right) radiating patches, and a defected ground plane. The single-element antenna sits on an FR4 substrate of 0.8 mm in thickness. The dielectric constant (*ε*_r_) and loss tangent (*tan*δ) of the substrate are 4.3 and 0.025. The single-element antenna is fed by an RG223/U coaxial cable connected to a 50 Ω SMA-type connector.

As shown in Fig. 1, the T-shaped hemispherical feeding patch is located between the left- and right-arm radiating patches separated by a coupling gap (i.e., coupling gap A). The coupling gap A is later optimized to maximize the impedance bandwidth of the single-element antenna and reduce the size of the antenna element.

The length of the T-shaped hemispherical feeding patch is equal to *F*_1_ + 2.8 mm + *F*_2_, where *F*_1_ and *F*_2_ are the long- and short-section shoulders of the T-shaped feeding patch. The length of the hemispherical head of the feeding patch (2.8 mm) is optimized by using CST Studio Suite. In the antenna design, the transmission line is connected to the T-shaped feeding patch at point *D*. The dimensions of the long- and short-arm radiating patches are denoted by *L*_1_ × *W*_1_ and *L*_2_ × *W*_2_, respectively. The ground plane is of two conjoined triangles (conjoining at point *E*). The distance between points *D* and *E* is equal to coupling gap *B*.

Simulations are carried out to optimize the antenna parameters using CST Microwave Studio Suite^42^. Table 1 tabulates the optimal physical and electrical dimensions of the single-element antenna.

**Table 1:** The optimal dimensions of the proposed broadband twin-element MIMO antenna scheme.

Parameter	Description	Physical size (mm)	Electrical size at 4.5 GHz
*W*	Total width of element	4.20	0.063λ_C_
*L*	Total length of element	30.98	0.465λ_C_
*L*_1_	Length of the long-section radiating patch	12.90	0.193λ_C_
*L*_2_	Length of the short-section radiating patch	6.84	0.103λ_C_
*W*_1_	Width of the long-section radiating patch	1.57	0.024λ_C_
*W*_2_	Width of the short-section radiating patch	1.77	0.027λ_C_
*F*_1_	Length of the long-section T-shaped shoulder	3.87	0.058λ_C_
*F*_2_	Length of the short-section T-shaped shoulder	2.32	0.035λ_C_
*W*_F_	Width of the T-shaped shoulder	0.79	0.012λ_C_
*L*_T1_	Height of larger triangle of ground plane	18.41	0.276λ_C_
*L*_T2_	Height of smaller triangle of ground plane	10.79	0.162λ_C_
*T*_g_	Base of smaller and larger triangles	1.30	0.019λ_C_
*W*_g_	Expanded width of ground plane	0.42	0.006λ_C_
*g*_A_	Coupling gap* A*	0.23	0.003λ_C_
*g*_B_	Distance between feeding patch and ground plane	0.70	0.010λ_C_
*d*_A_	Edge-to-edge distance between the two antenna elements	98.79	1.482λ_C_
*d*_S_	Vertical distance between the reflector and the base of the antenna element	13.00	0.195λ_C_

*λ_C_ is the wavelength at the center frequency of the operating frequency band (3.5–5.5 GHz).

### Twin-Element Antenna Scheme with Laptop Model

**Figure 2:**
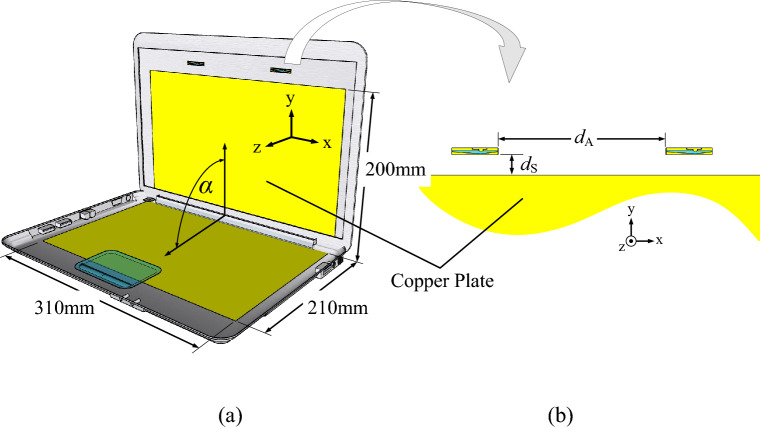
Geometry of the twin-element MIMO antenna scheme integrated with the laptop model: (**a**) perspective view, (**b**) side view.

Figure 2a shows a laptop model integrated with two single-element antennas. The laptop model consists of vertical and horizontal slats perpendicular to each other (α = 90°), and the two single-element antennas constitute the twin-element MIMO antenna scheme. In this research, the laptop is used as the reflector for unidirectional radiation pattern. The optimal edge-to-edge distance between the two antenna elements (*d*_A_) is 98.79 mm, with mutual coupling (|*S*_12_|) less than − 40 dB. To reduce the back lobe level, the optimal vertical distance between the reflector and the base of the antenna element (*d*_S_) is 13 mm (Fig. 2b).

## Parametric Study

### Evolution of Single-Element Antenna

**Figure 3:**
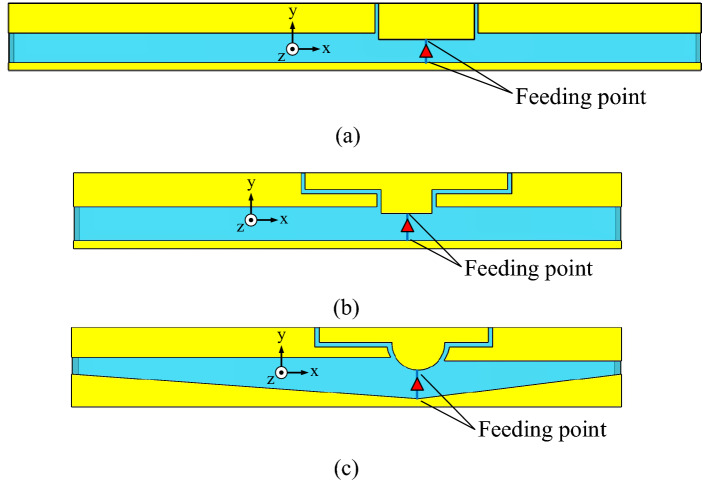
Evolutionary stages of the single-element antenna: (**a**) first generation, (**b**) second generation, (**c**) third generation.

The evolution of the single-element antenna involves three steps: first-, second-, and third-generation antennas, as shown Fig. 3a–c. The first-generation antenna is of dual-arm antenna structure with a rectangular feeding patch separated by a coupling gap. The simulation results demonstrate that the first-generation antenna fails to cover the entire operating frequency band (i.e., 3.5–5.5 GHz). Specifically, the first-generation antenna suffers from a narrow impedance bandwidth, covering 3.8–5.0 GHz.

In the second-generation antenna, the rectangular feeding patch is transformed into a T-shaped feeding patch to improve the impedance bandwidth. In addition, the length of the coupling gap is extended. However, the second-generation antenna still fails to cover the entire operating frequency band, covering 3.4–5.0 GHz.

In the third-generation antenna, the T-shaped feeding patch is reshaped into a hemispherical T-shaped feeding patch, and the ground plane is reconfigured into conjoined triangles (i.e., defected ground plane). The third-generation antenna covers the entire operating frequency band, covering 3.4–5.6 GHz.

**Figure 4:**
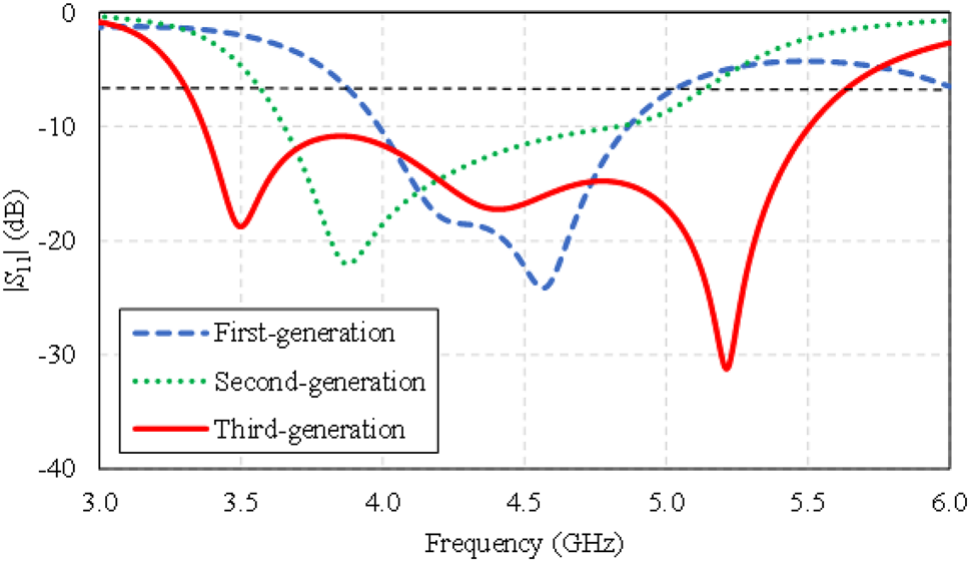
Simulated impedance bandwidths (|*S*_11_|≤ − 6 dB) of the first-, second-, and third-generation single-element antennas.

**Figure 5:**
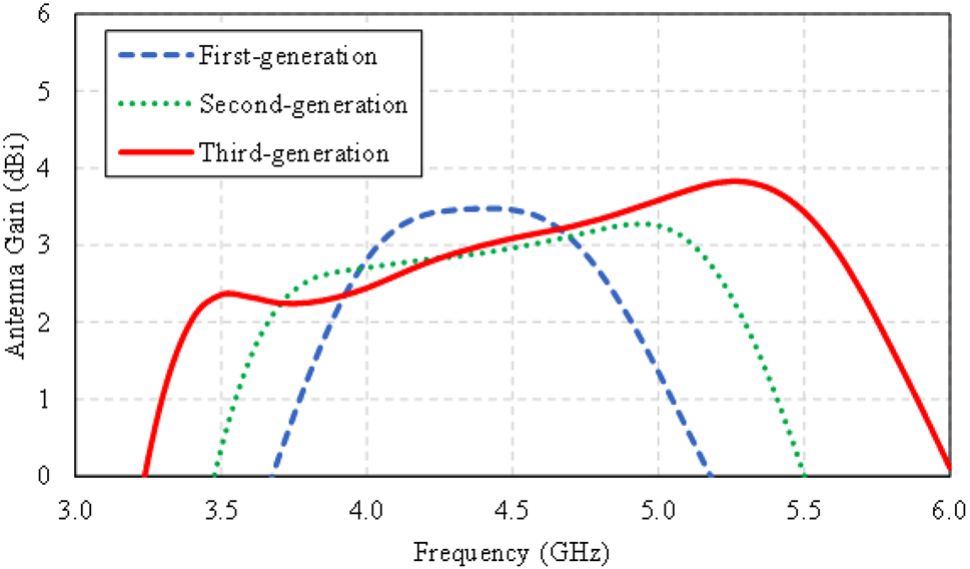
Simulated antenna gains of the first-, second-, and third-generation single-element antennas.

Figure 4 compares the simulated impedance bandwidths (|*S*_11_|≤ − 6 dB) of the first-, second-, and third-generation single-element antennas. The first- and second-generation antennas have a narrow impedance bandwidth, covering 3.8–5.0 GHz and 3.4–5.0 GHz, respectively. The third-generation antenna achieves a wider impedance bandwidth, covering 3.4–5.6 GHz. Figure 5 shows the simulated antenna gains of the first-, second-, and third-generation single-element antennas.

**Figure 6:**
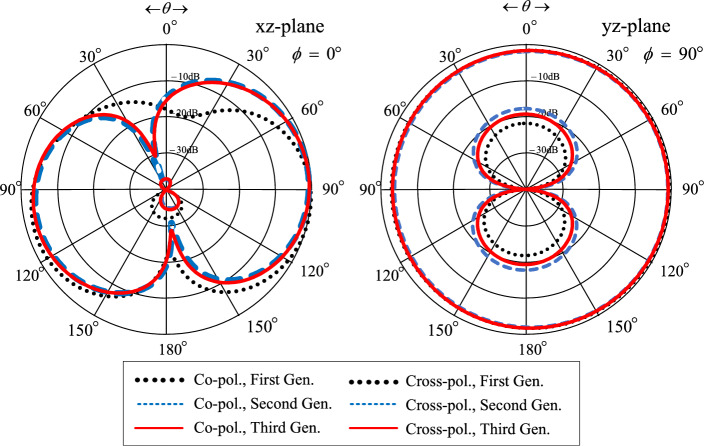
Simulated xz- and yz-plane radiation patterns of the first-, second-, and third-generation single-element antennas at the center frequency of 4.5 GHz.

Figure 6 shows the simulated xz- and yz-plane radiation patterns of the first-, second-, and third-generation single-element antennas. Although the radiation patterns of the three antennas closely resemble one another, only the third-generation antenna can cover the entire operating frequency band (3.5–5.5 GHz). As a result, the third-generation single-element antenna is selected for subsequent experiment.

**Figure 7:**
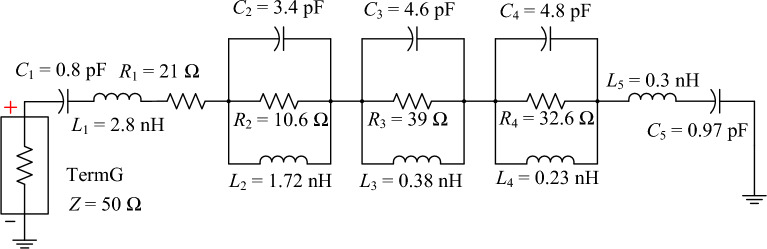
Equivalent circuit model of the third-generation single-element antenna.

Figure 7 illustrates the equivalent circuit model of the third-generation single-element antenna to determine the impedance matching and bandwidth across the operating frequency band. The equivalent circuit model consists of a series *RLC*, three parallel *RLC* circuits, and a series *LC* component. The initial values of the equivalent circuit elements are calculated by^43^, and the optimized component values are determined by using ADS software.

**Figure 8:**
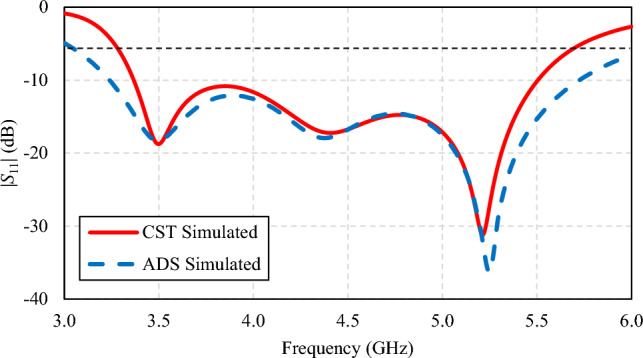
Comparison between the simulated impedance bandwidths (|*S*_11_|≤ − 6 dB) of the third-generation single-element antenna by CST and by Advanced Design System (ADS).

**Figure 9:**
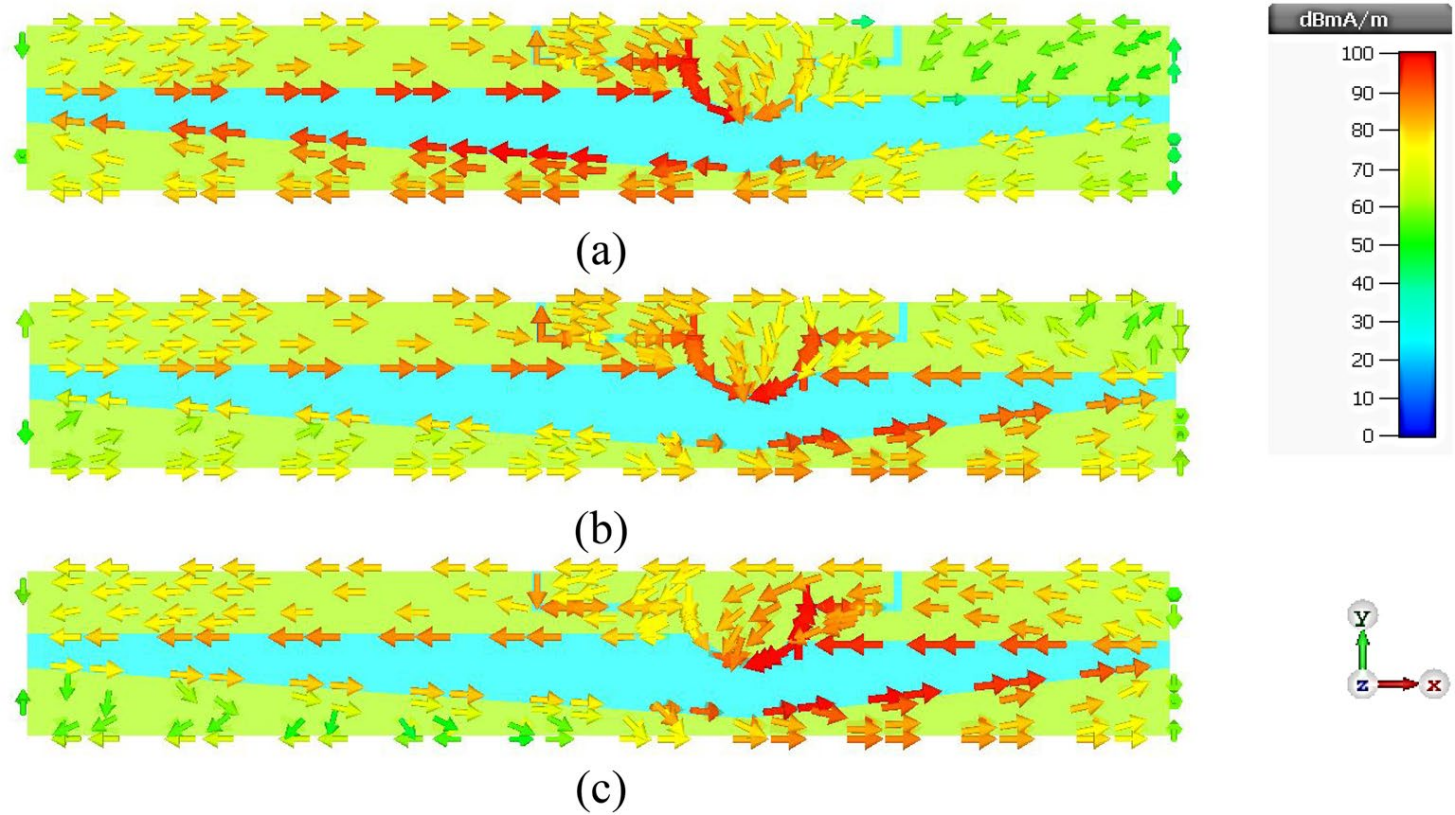
Simulated surface current distribution of the twin-element MIMO antenna scheme with the first antenna element excited and the second antenna element terminated: (**a**) 3.5 GHz, (**b**) 4.5 GHz, (**c**) 5.5 GHz.

**Figure 10:**
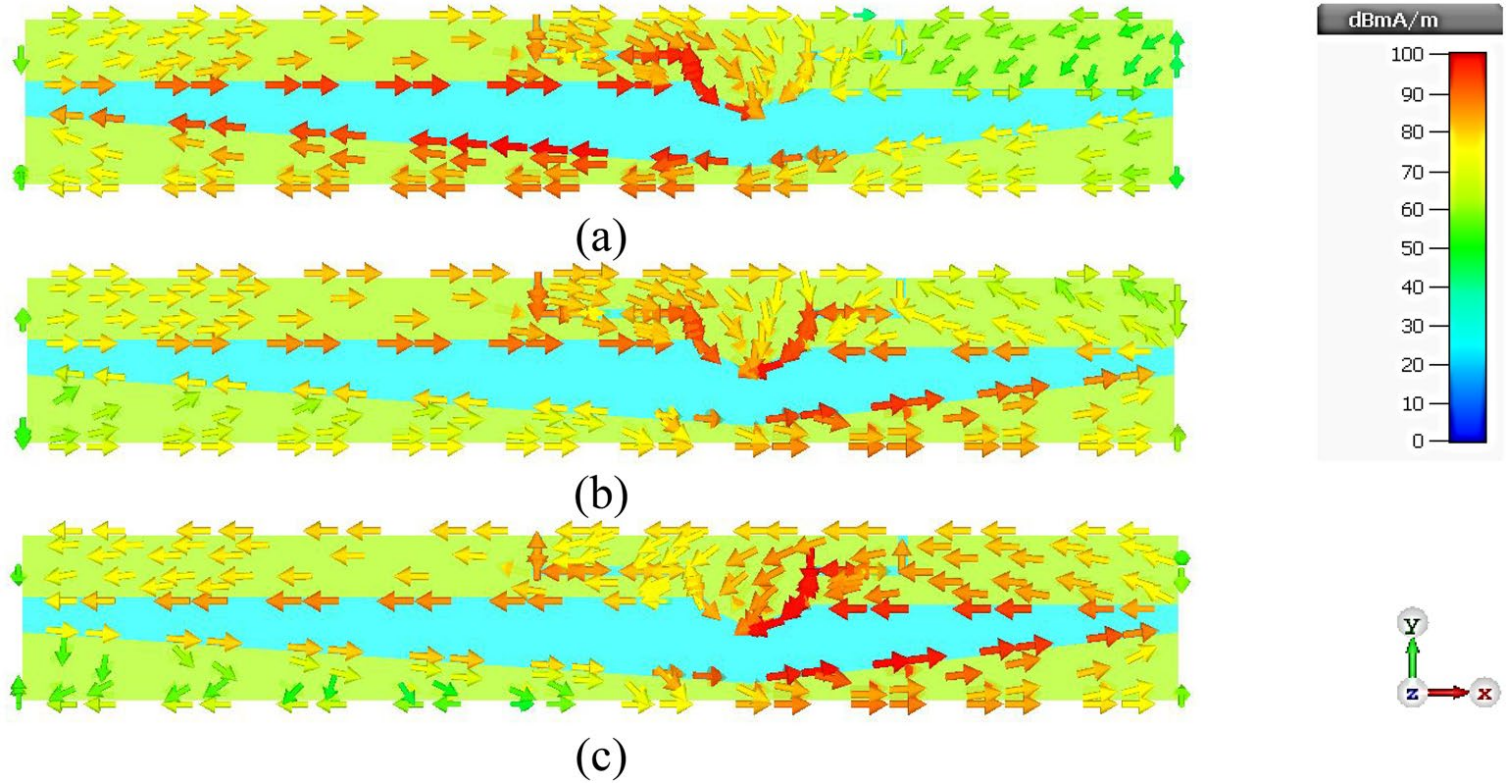
Simulated surface current distribution of the twin-element MIMO antenna scheme with the first antenna element terminated and the second antenna element excited: (**a**) 3.5 GHz, (**b**) 4.5 GHz, (**c**) 5.5 GHz.

Figure 8 compares the simulated impedance bandwidths (|*S*_11_|≤ − 6 dB) of the third-generation single-element antenna by CST and by ADS. In the figure, the first, second, and third parallel *RLC* circuits resonate at 3.5, 4.4, and 5.25 GHz, respectively, corresponding to the surface current distribution in Figs. 9 and 10. Both CST and ADS models achieve wide impedance bandwidth across the operating frequency band of 3.5 to 5.5 GHz.

### Surface Current Distribution of Twin-Element MIMO Antenna Scheme

The surface current distribution of the twin-element MIMO antenna scheme is simulated by exciting one element and terminating the other element using a 50 Ω load. The twin-element MIMO antenna scheme comprises two elements of the third-generation antenna due to the wide impedance bandwidth and high gain characteristics (Figs. 4 and 5).

Figure 9a–c show the surface current distribution of the twin-element MIMO antenna scheme at 3.5, 4.5, and 5.5 GHz, with the 1^st^ antenna element excited and the 2^nd^ antenna element terminated. At 3.5 GHz, the surface current flows along the left-shoulder of the feeding patch, the left-arm radiating patch, and the larger triangular ground plane. At 5.5 GHz, the surface current flows along the right-shoulder of the feeding patch, the right-arm radiating patch, and the smaller triangular ground plane. Specifically, the left-arm radiating patch resonates at the lower frequency of 3.5 GHz, while the right-arm radiating patch resonates at the higher frequency of 5.5 GHz. At 4.5 GHz, the surface current is concentrated around the head of the feeding patch. Given the perfect symmetry of the twin-element antenna scheme, the same behavior is observed when the 1^st^ antenna element is terminated while the 2^nd^ antenna element is excited, as shown in Fig. 10a–c. The mutual coupling (|*S*_12_|) between the two antenna elements is less than − 40 dB with negligible current leakage.

### Parametric Study of Twin-Element MIMO Antenna Scheme

This section examines the effects of antenna parameters on the impedance bandwidth (|*S*_11_|, |*S*_22_|≤ − 6 dB) and mutual coupling (|*S*_12_|) of the proposed twin-element MIMO antenna scheme, as shown in Figs. 11, 12, 13, 14, 15, 16, 17 and 19, 20.

**Figure 11:**
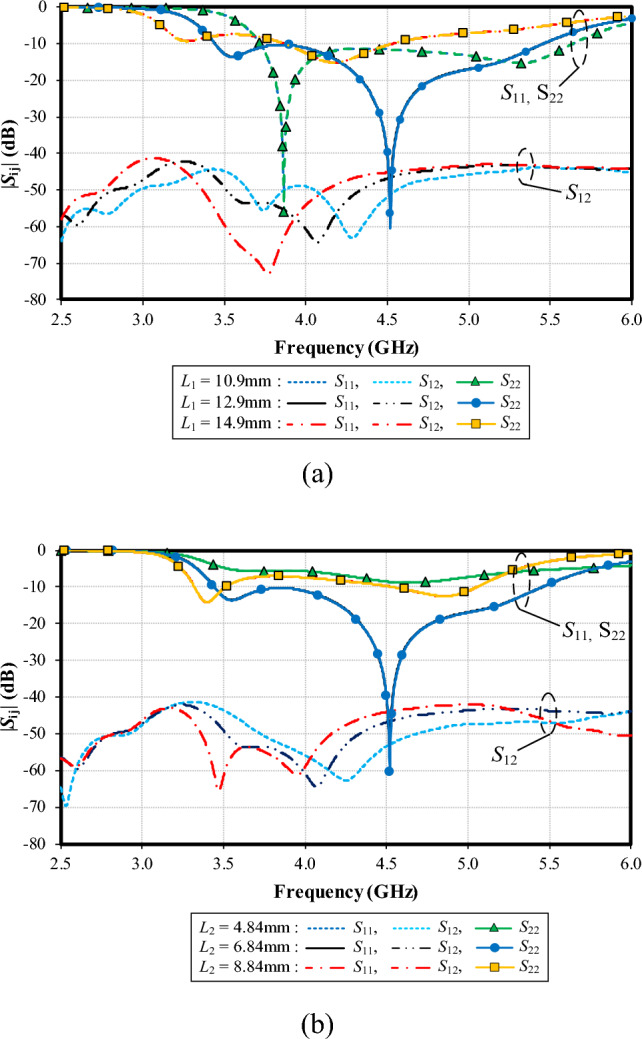
Simulated impedance bandwidth (|*S*_11_|, |*S*_22_|≤ − 6 dB) and mutual coupling (|*S*_12_|) under variable lengths of left- (*L*_1_) and right-arm radiating patches (*L*_2_): (**a**) *L*_1_, (**b**) *L*_2_.

Figure 11a shows the simulated impedance bandwidth (|*S*_11_|, |*S*_22_|≤ − 6 dB) and mutual coupling (|*S*_12_|) under variable *L*_1_: 10.9, 12.9, and 14.9 mm. With *L*_1_ = 10.9 mm, the resonance frequency is at 3.8 GHz, with the impedance matching of − 54 dB. With *L*_1_ = 12.9 mm, the resonance frequency is at 4.5 GHz (− 60 dB). Given *L*_1_ = 14.9 mm, the resonance frequency is at 4.25 GHz (− 15 dB). The optimal *L*_1_ is thus 12.9 mm. The mutual coupling (|*S*_12_|) is less than − 40 dB for the entire operating frequency band, independent of *L*_1_.

Figure 11b shows the simulated |*S*_11_|, |*S*_22_| and |*S*_12_| under variable *L*_2_: 4.84, 6.84, and 8.84 mm. Given* L*_2_ = 6.84 mm, the lowest impedance matching of − 60 dB is achieved at 4.5 GHz (i.e., the resonance frequency). The optimal *L*_2_ is 6.84 mm. The mutual coupling (|*S*_12_|) is less than − 40 dB for the entire operating frequency band, independent of *L*_2_.

**Figure 12:**
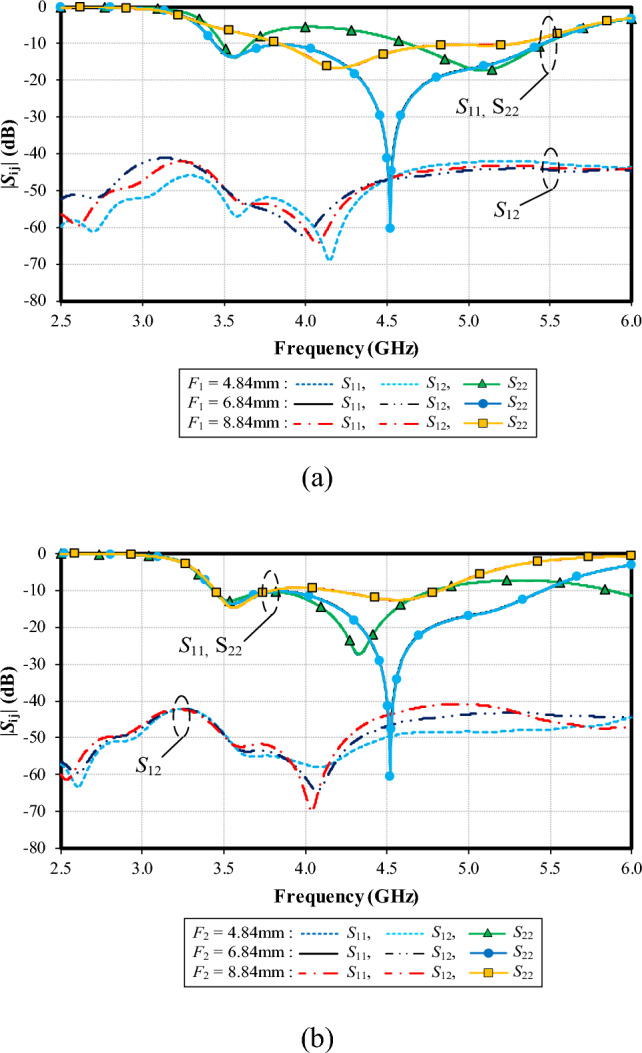
Simulated impedance bandwidth (|*S*_11_|, |*S*_22_|≤ − 6 dB) and mutual coupling (|*S*_12_|) under variable lengths of the left (*F*_1_) and right shoulders of the feeding patch (*F*_2_): (**a**) *F*_1_, (**b**) *F*_2_.

Figure 12a shows the simulated |*S*_11_|, |*S*_22_| and |*S*_12_| under variable lengths of the left shoulder of the feeding patch (*F*_1_): 1.87, 3.87, and 5.87 mm. Given *F*_1_ = 3.87 mm, the lowest impedance matching of − 60 dB is achieved at 4.5 GHz. |*S*_12_| is below − 40 dB for the entire operating frequency band, independent of *F*_1_.

Figure 12b shows the simulated |*S*_11_|, |*S*_22_| and |*S*_12_| under variable lengths of the right shoulder of the feeding patch (*F*_2_): 0.32, 2.32, and 4.32 mm. With *F*_2_ = 0.32 mm, the resonance frequency is at 4.3 GHz, with the impedance matching of − 28 dB. With *F*_2_ = 2.32 mm, the resonance frequency is at 4.5 GHz (− 60 dB). With *F*_2_ = 4.32 mm, the resonance frequency is at 3.6 GHz (− 15 dB). The optimal *F*_2_ is 2.32 mm. |*S*_12_| is less than − 40 dB for the entire operating frequency band, independent of *F*_2_.

**Figure 13:**
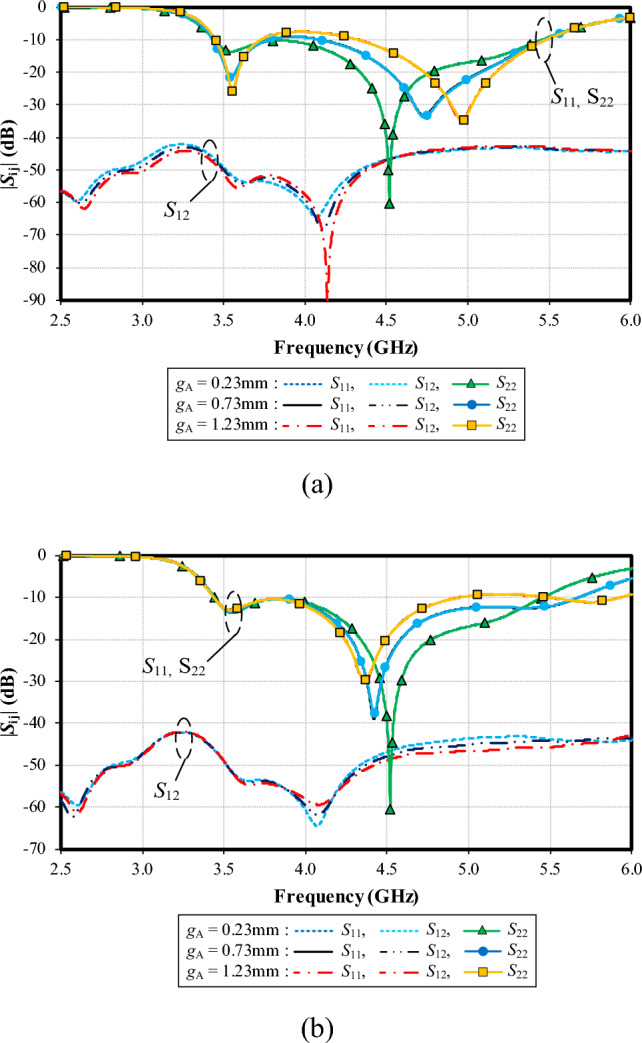
Simulated impedance bandwidth (|*S*_11_|, |*S*_22_|≤ − 6 dB) and mutual coupling (|*S*_12_|) of the left and right shoulders of the feeding patch under variable width of coupling gap (*g*_A_): (**a**) the left (long) shoulder, (**b**) the right (short) shoulder.

Figure 13a, b show the simulated |*S*_11_|, |*S*_22_| and |*S*_12_| of the left and right shoulders of the feeding patch under variable widths of coupling gap (*g*_A_): 0.23, 0.73, and 1.23 mm. In Fig. 11a (for the left shoulder of the feeding patch), with *g*_A_ = 0.23 mm, the resonance frequency is at 4.5 GHz, with the impedance matching of − 60 dB. With *g*_A_ = 0.73 mm, the resonance frequency is at 4.75 GHz (− 32 dB) and at 5 GHz (− 35 dB) for *g*_A_ = 1.23 mm.

In Fig. 13b (for the right shoulder of the feeding patch), with *g*_A_ = 0.23 mm, the resonance frequency is at 4.5 GHz, with the impedance matching of − 60 dB. With *g*_A_ = 0.73 mm, the resonance frequency is at 4.4 GHz (− 40 dB) and at 4.35 GHz (− 30 dB) for *g*_A_ = 1.23 mm. The optimal *g*_A_ is thus 0.23 mm, achieving the lowest impedance matching (− 60 dB) at the center frequency of 4.5 GHz for the left and right shoulders of the feeding patch.

**Figure 14:**
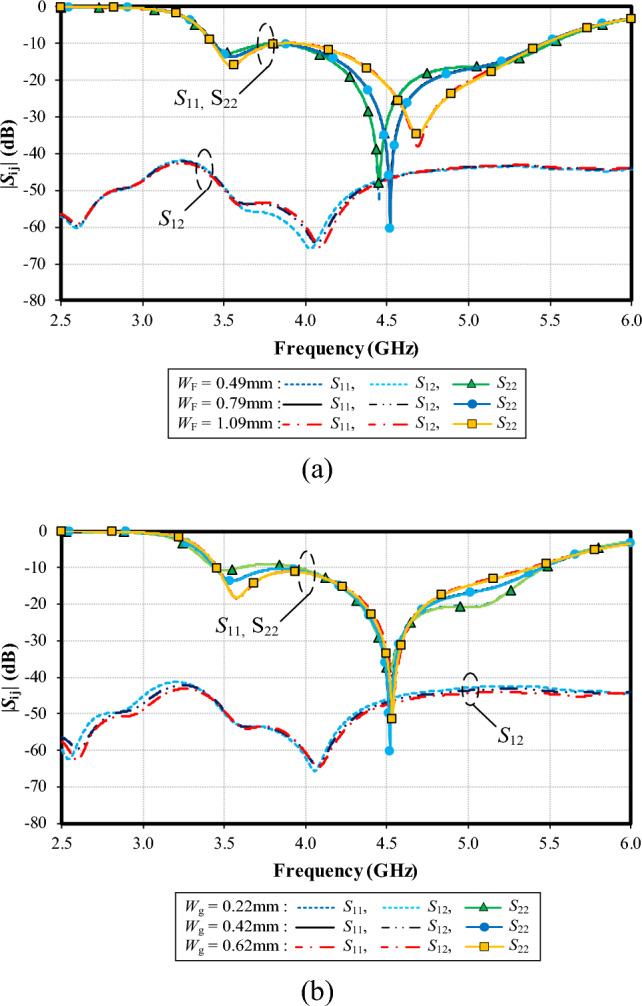
Simulated impedance bandwidth (|*S*_11_|, |*S*_22_|≤ − 6 dB) and mutual coupling (|*S*_12_|) under variable *W*_f_ and *W*_g_: (**a**) width of the T-shaped shoulder (*W*_f_), (**b**) expanded width of ground plane (*W*_g_).

Figure 14a shows the simulated |*S*_11_|, |*S*_22_| and |*S*_12_| under variable width of the T-shaped shoulder (*W*_f_): 0.49, 0.79, and 1.09 mm. With *W*_f_ = 0.49 mm, the resonance frequency is at 4.45 GHz, with the impedance matching of − 50 dB, and the resonance frequency is at 4.5 GHz (− 60 dB) for *W*_f_ = 0.79 mm. Given *W*_f_ = 1.09 mm, the resonance frequency is at 4.65 GHz (− 38 dB). The optimal *W*_f_ is 0.79 mm. The mutual coupling (|*S*_12_|) is below − 40 dB for the entire operating frequency band, independent of *W*_f_.

Figure 14b shows the simulated |*S*_11_|, |*S*_22_| and |*S*_12_| under variable width of the expanded width of ground plane (*W*_g_): 0.22, 0.42, and 0.62 mm. The resonance frequency is almost identical (at 4.5 GHz), independent of *W*_g_. However, the lowest impedance matching of − 60 dB is achieved with *W*_g_ = 0.42. Therefore, the optimal *W*_g_ is 0.42.

**Figure 15:**
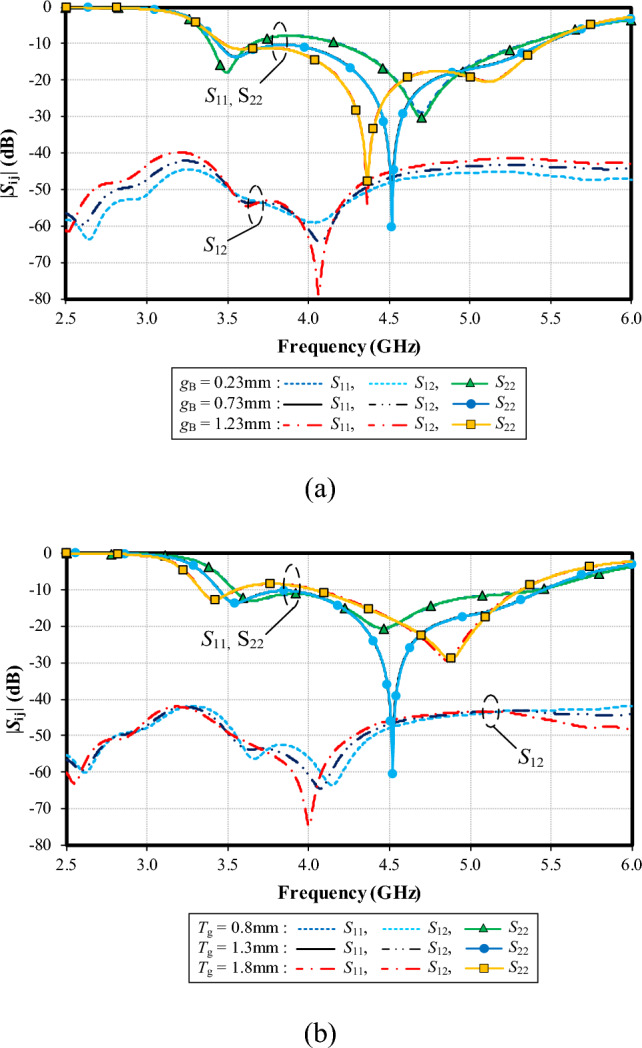
Simulated impedance bandwidth (|*S*_11_|, |*S*_22_|≤ − 6 dB) and mutual coupling (|*S*_12_|) under variable *g*_B_ and *T*_g_: (**a**) distance between the feeding patch and ground plane (*g*_B_), (**b**) height of the base of small- and large- triangle ground plane (*T*_g_).

Figure 15a shows the simulated |*S*_11_|, |*S*_22_| and |*S*_12_| under variable width between the feeding patch and ground plane (*g*_B_): 1.01, 1.51, and 2.01 mm. With *g*_B_ = 1.01 mm, the resonance frequency is at 4.7 GHz, with the impedance matching of − 30 dB, and the resonance frequency is at 4.5 GHz (− 60 dB) for *g*_B_ = 1.51 mm. Given *g*_B_ = 2.01 mm, the resonance frequency is at 4.3 GHz (− 50 dB). The optimal *g*_B_ is 1.51 mm. The mutual coupling (|*S*_12_|) is below − 40 dB for the entire operating frequency band, independent of *g*_B_.

Figure 15b shows the simulated |*S*_11_|, |*S*_22_| and |*S*_12_| under variable height of the base of small- (right) and large-triangle (left) ground plane (*T*_g_): 0.80,1.30, and 1.80 mm. Given *T*_g_ = 1.30 mm, the resonance frequency is at the center frequency of 4.5 GHz, achieving the lowest impedance matching of − 60 dB. The optimal *T*_g_ is 1.30 mm. The mutual coupling |*S*_12_| is below − 40 dB for the entire operating frequency band, independent of *T*_g_.

**Figure 16:**
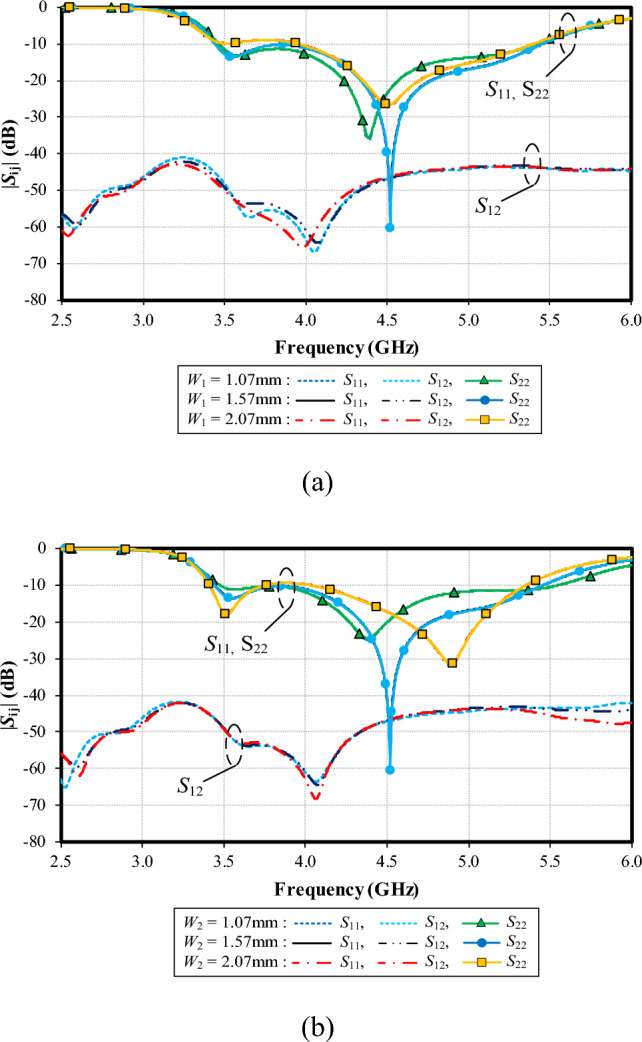
Simulated impedance bandwidth (|*S*_11_|, |*S*_22_|≤ − 6 dB) and mutual coupling (|*S*_12_|) under variable width of the left- (*W*_1_) and right-arm radiating patches (*W*_2_): (a) *W*_1_, (b) *W*_2_.

Figure 16a shows the simulated |*S*_11_|, |*S*_22_| and |*S*_12_| under variable width of the left-arm radiating patch (*W*_1_): 1.07, 1.57, and 2.07 mm. With *W*_1_ = 1.07 mm, the resonance frequency is at 4.4 GHz, with the impedance matching of − 35 dB, and the resonance frequency is at 4.5 GHz (− 60 dB) for *W*_1_ = 1.57 mm. Given *W*_1_ = 2.07 mm, the resonance frequency is at 4.5 GHz (− 28 dB). The optimal *W*_1_ is 1.57 mm. The mutual coupling (|*S*_12_|) is below − 40 dB for the entire operating frequency band, independent of *W*_1_.

Figure 16b shows the simulated |*S*_11_|, |*S*_22_| and |*S*_12_| under variable width of the right-arm radiating patch (*W*_2_): 1.27, 1.77, and 2.27 mm. With *W*_2_ = 1.27 mm, the resonance frequency is at 4.35 GHz, with the impedance matching of − 25 dB, and the resonance frequency is at 4.5 GHz (− 60 dB) for *W*_2_ = 1.77 mm. Given *W*_2_ = 2.27 mm, the resonance frequency is at 4.8 GHz (− 30 dB). The optimal *W*_2_ is 1.77 mm. The mutual coupling (|*S*_12_|) is less than − 40 dB for the entire operating frequency band, independent of *W*_2_.

**Figure 17:**
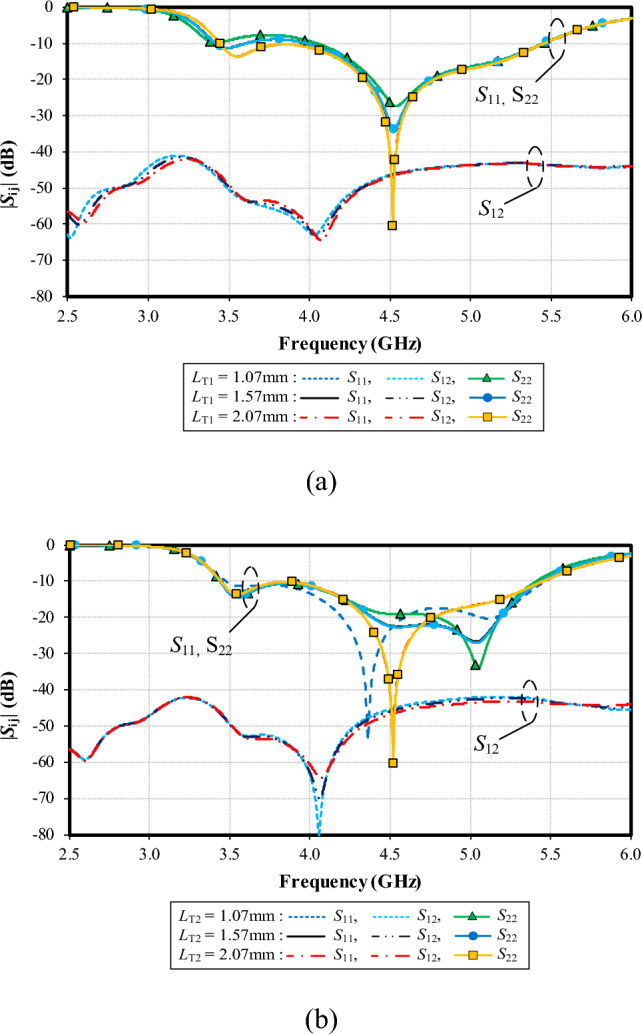
Simulated impedance bandwidth (|*S*_11_|, |*S*_22_|≤ − 6 dB) and mutual coupling (|*S*_12_|) under variable length of large- (left) (*L*_T1_) and small-triangular (right) ground plane (*L*_T2_): (**a**) *L*_T1_, (**b**) *L*_T2_.

Figure 17a shows the simulated |*S*_11_|, |*S*_22_| and |*S*_12_| under variable length of the larger triangle of the ground plane (*L*_T1_): 14.41, 16.41, and 18.41 mm. The resonance frequency is almost identical (at 4.5 GHz), independent of *L*_T1_. However, the lowest impedance matching of − 60 dB is achieved with *L*_T1_ = 18.41. Therefore, the optimal *L*_T1_ is 18.41. The mutual coupling (|*S*_12_|) is below − 40 dB for the entire operating frequency band, independent of *L*_T1_.

Figure 17b shows the simulated |*S*_11_|, |*S*_22_| and |*S*_12_| under variable length of the smaller triangle of the ground plane (*L*_T2_): 6.79, 8.79, and 10.79 mm. With *L*_T2_ = 6.79 mm, the resonance frequency is at 5.1 GHz, with the impedance matching of − 32 dB, and the resonance frequency is at 5.1 GHz (− 25 dB) for *L*_T2_ = 8.79 mm. Given *L*_T2_ = 10.79 mm, the resonance frequency is at 4.5 GHz (− 60 dB). The optimal *L*_T2_ is 10.79 mm. The mutual coupling (|*S*_12_|) is below − 40 dB for the entire operating frequency band, independent of *L*_T2_.

**Figure 18:**
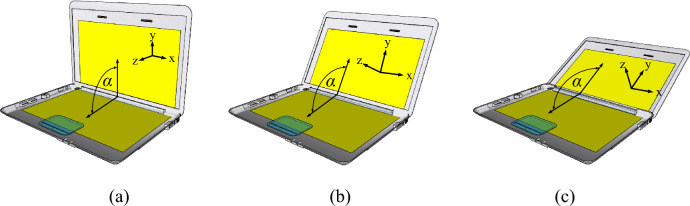
The angle (α) between the horizontal and vertical slats of the laptop model: (**a**) 90°, (**b**) 120°, (**c**) 150°.

**Figure 19:**
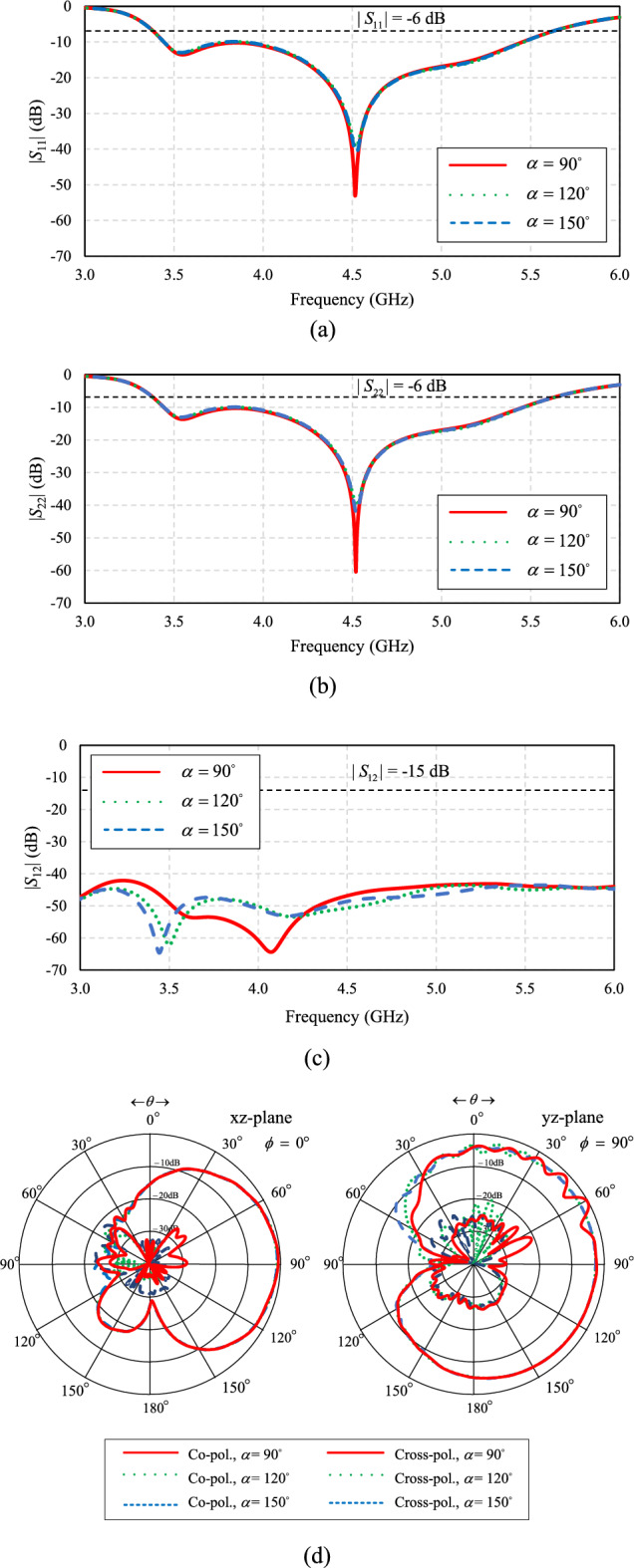
The simulated results of the proposed twin-element MIMO antenna scheme integrated with the laptop model at variable angles between the horizontal and vertical slats: (**a**) |*S*_11_|, (**b**) |*S*_22_|, (**c**) |*S*_12_|, (**d**) radiation pattern.

Figure 18a–c depict the laptop model at variable angles (α) of 90°, 120°, and 150°, respectively. Figure 19a–d show the corresponding simulated |*S*_11_|, |*S*_22_|, |*S*_12_|, and radiation pattern of the proposed twin-element MIMO antenna scheme integrated with the laptop model.

In Fig. 19a, b, the simulated |*S*_11_| and |*S*_22_| are less than − 6 dB across the operating frequency band, independent of α. The results are consistent with^43^, indicating that the angle (α) of the laptop model has no impact on the impedance bandwidth of the antenna. In Fig. 19c, given α = 90°, the lowest |*S*_12_| is closest to the center frequency of 4.5 GHz. In Fig. 19d, the radiation patterns closely resemble one another, independent of α.

**Figure 20:**
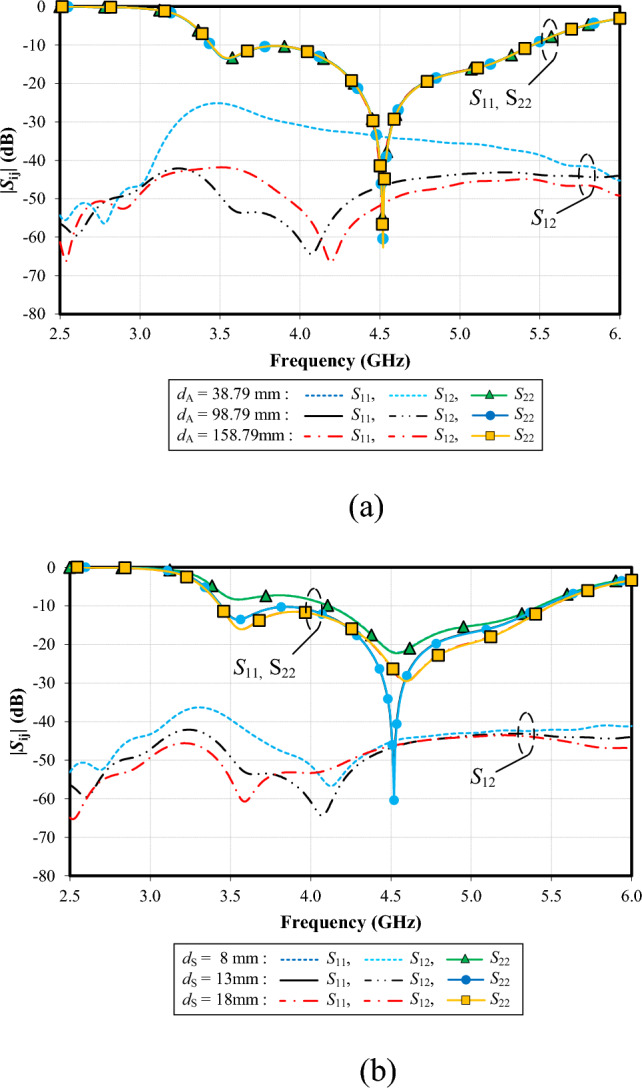
Simulated impedance bandwidth (|*S*_11_|, |*S*_22_|≤ − 6 dB) and mutual coupling (|*S*_12_|) of the proposed twin-element MIMO antenna scheme integrated with the laptop model under variable edge-to-edge distance between the two antenna elements (*d*_A_) and vertical distance between the reflector and the base of the antenna element (*d*_S_): (**a**) *d*_A_, (**b**) *d*_S_.

Figure 20a shows the simulated |*S*_11_|, |*S*_22_| and |*S*_12_| under variable edge-to-edge distance between the two antenna elements (*d*_A_): 38.79, 98.79, and 158.79 mm. The resonance frequency is almost identical (at 4.5 GHz), independent of *d*_A_. The mutual coupling (|*S*_12_|) is higher than − 40 dB between 3.1 and 5.5 GHz for *d*_A_ = 38.79 mm. In contrast, the mutual coupling (|*S*_12_|) is below − 40 dB for the entire operating frequency band for *d*_A_ = 98.79 mm and 158.79 mm. Due to its smaller distance, *d*_A_ = 98.79 mm is the optimal choice.

Figure 20b shows the simulated |*S*_11_|, |*S*_22_| and |*S*_12_| under variable vertical distance between the reflector and the base of the antenna element (*d*_S_): 8, 13, and 18 mm. The resonance frequency is almost identical (at 4.5 GHz), independent of *d*_S_. However, the lowest impedance matching of − 60 dB is achieved with *d*_S_ = 13 mm. Therefore, the optimal *d*_S_ is 13 mm. The mutual coupling (|*S*_12_|) exceeds − 40 dB between 3.1 and 3.5 GHz for *d*_S_ = 8 mm. With *d*_S_ = 13 mm and 18 mm, the mutual coupling (|*S*_12_|) remains below − 40 dB across the entire frequency band. Given its smaller distance, *d*_S_ of 13 mm is selected.

## Prototype Fabrication and Measured Results

**Figure 21:**
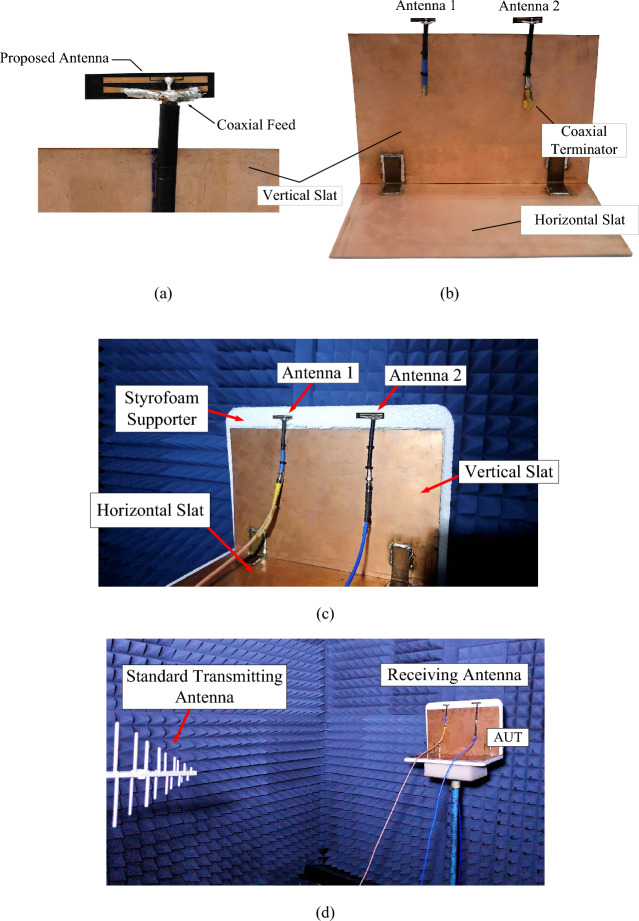
Prototype antenna: (**a**) single-element antenna, (**b**) the twin-element MIMO antenna scheme integrated with horizontal and vertical slats, (**c**) the twin-element MIMO antenna scheme integrated with horizontal and vertical slats with Styrofoam supporter, (**d**) antenna measurement in an anechoic chamber.

In this research, each single-element antenna of the twin-element MIMO antenna scheme is designed to operate independently. Figure 21a,b show the prototype of a single-element antenna on FR4 substrate. Figure 21c illustrates the twin-element MIMO antenna scheme integrated with the laptop model. The twin-element antenna scheme consists of two single-element antennas, and the distance between both single-element antennas (*d*_A_) is 98.79 mm. The antenna elements are fed by the coaxial cables of 8 cm in length without bends, as illustrated in Fig. 21b. Shorter coaxial cables with minimal bends enhances the antenna performance and minimizes losses and attenuation, compared with longer coaxial cables^44^. The laptop model is of the horizontal and vertical copper slats angled at 90°. The laptop model is used as the reflector.

In the experiment, to characterize the transmission characteristics of the twin-element MIMO antenna scheme, an antenna under test (AUT), as the receiving antenna, was positioned in the middle of the anechoic chamber. A motorized positioning system rotated the AUT around the horizontal and vertical axes. A log-periodic antenna (Model USLP 9143, covering 300 MHz–5 GHz) was used as the transmitting antenna, and the measurement was conducted using Agilent E5061B network analyzer, as illustrated in Fig. 21d. The radiation patterns of the twin-element MIMO antenna scheme are measured in the xz and yz planes. The distance between the transmitting log-periodic antenna and the tested twin-element MIMO antenna scheme is greater than the far-field distance. The boresight gain is measured and calculated by the Friis transmission formula.

**Figure 22:**
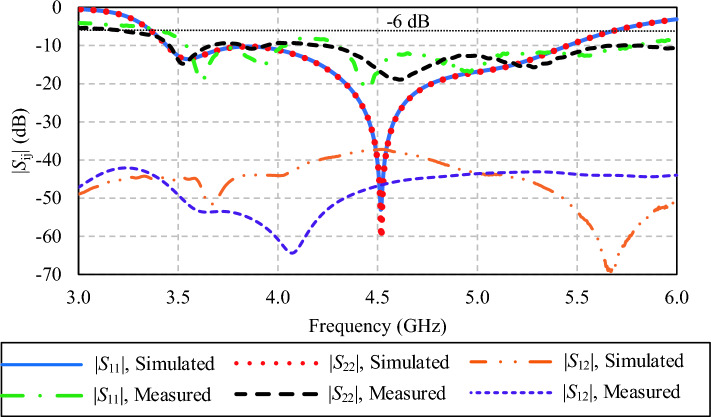
Simulated and measured |*S*_11_|, |*S*_22_|, and |*S*_12_| of the twin-element MIMO antenna scheme with the laptop model.

Figure 22a–c show the simulated and measured |*S*_11_|, |*S*_22_| and |*S*_12_| of the proposed twin-element MIMO antenna scheme integrated with the laptop model. The simulated |*S*_11_| and |*S*_22_| are 51.50% (3.36–5.69 GHz), and the measured results are 55.32%, covering 3.4–6.0 GHz. The simulated and measured |*S*_12_| are below − 15 dB, indicating no interference between the two single-element antennas. Discrepancies between the simulated and measured results for the reflection coefficients and mutual coupling could be attributed to the controlled parameters in the simulations, whereas the measurement is susceptible to fabrication imperfections, insertion loss, and signal reflections.

**Figure 23:**
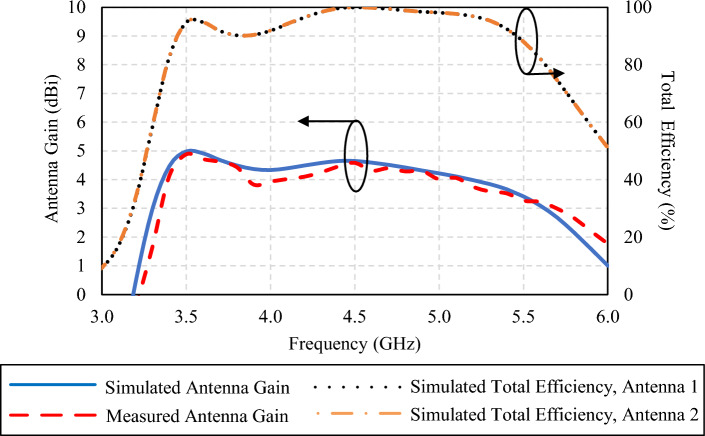
Simulated and measured antenna gain and total antenna efficiency of the twin-element MIMO antenna scheme with the laptop model.

In Fig. 23, the simulated gains of the proposed twin-element MIMO antenna scheme integrated with the laptop model at 3.5, 4.5, and 5.5 GHz are 4.98, 4.65, and 3.42 dBi, respectively. The corresponding measured gains are 4.878, 4.585, and 3.265 dBi. The simulated and measured antenna gains are agreeable. The simulated total antenna efficiency is between 90 and 100% across the operating frequency band. This level of efficiency indicates that the proposed antenna scheme can effectively convert input power into radiated power, resulting in minimal losses and optimal performance across the target operating frequency range.

In Figs. 22 and 23, the larger discrepancies between the simulated and measured reflection coefficients are probably attributable to higher susceptibility to environmental and/or setup variations of the reflection coefficients, in comparison with the antenna gains^45^.

**Figure 24:**
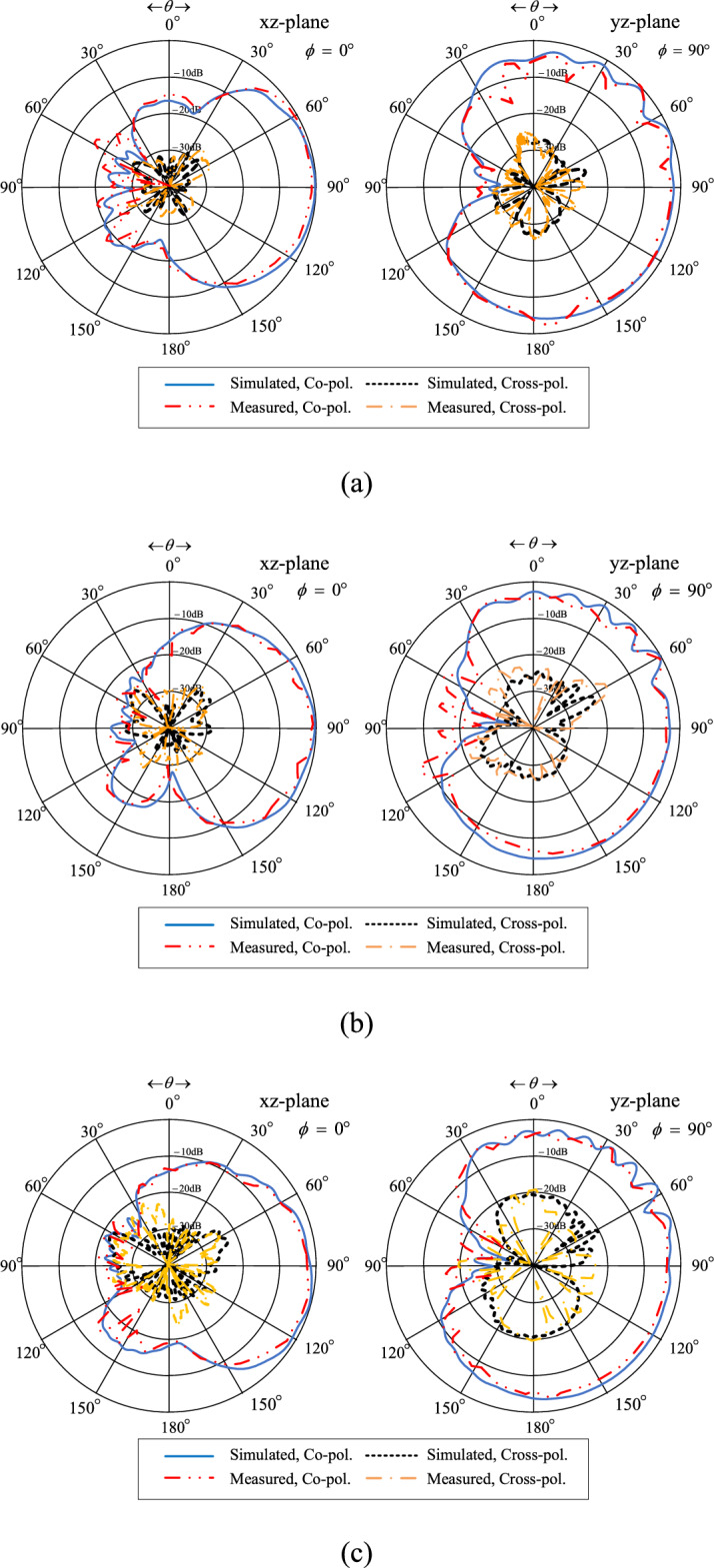
Simulated and measured xz- and yz-plane radiation patterns of the twin-element MIMO antenna scheme with the laptop model: (**a**) 3.5 GHz, (**b**) 4.5 GHz, (**c**) 5.5 GHz.

Figure 24a–c show the simulated and measured xz- and yz-plane radiation patterns of the twin-element MIMO antenna scheme integrated with the laptop model at 3.5, 4.5, and 5.5 GHz, respectively. The radiation pattern of the proposed twin-element MIMO antenna scheme is of unidirectionality.

The measured xz- and yz-plane cross-polarization levels are below − 25 dB and − 15 dB, respectively. The measured half-power beamwidth (HPBW) in the xz-plane at 3.5, 4.5, and 5.5 GHz are 99°, 92.8°, and 84.2°. The corresponding HPBW in the yz-plane are 102°, 78°, and 102°. The measured xz- and yz-plane back lobe levels are below − 15 dB across the entire operating frequency band.

**Figure 25:**
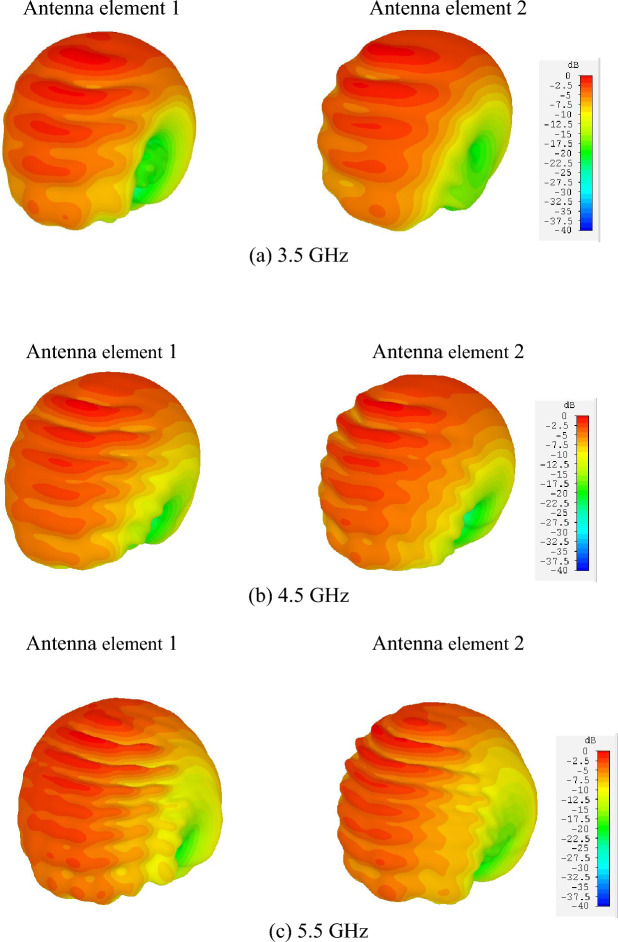
Simulated 3D radiation patterns of the twin-element MIMO antenna scheme with the laptop model: (**a**) 3.5 GHz, (**b**) 4.5 GHz, (**c**) 5.5 GHz.

Figure 25a–c illustrate the simulated 3D radiation patterns of the twin-element MIMO antenna scheme with the laptop model at 3.5, 4.5, and 5.5 GHz. Table 2 tabulates the simulated and measured performance of the proposed broadband unidirectional twin-element MIMO antenna scheme. Table 3 compares between the proposed broadband unidirectional twin-element MIMO antenna scheme and existing research works.

**Table 2:** Simulated and measured performance of the broadband unidirectional twin-element MIMO antenna scheme.

Items	Simulation			Measurement		
	3.5 GHz	4.5 GHz	5.5 GHz	3.5 GHz	4.5 GHz	5.5 GHz
|*S*_11_|≤ − 6 dB bandwidth, %	(3.36–5.69), 51.50%			(3.40–6.0), 55.32%		
|*S*_22_|≤ − 6 dB bandwidth, %	(3.36–5.69), 51.50%			(3.40–6.0), 55.32%		
Antenna gain (dBi)	4.98	4.65	3.42	4.878	4.585	3.265
HPBW in xz-plane, (deg.)	149	194	185	99	92.8	84.2
HPBW in yz-plane, (deg.)	150	159	160	102	78	102
Cross-pol. in xz-plane (dB)	≤ − 20			≤ − 25		
Cross-pol. in yz-plane (dB)	≤ − 20			≤ − 15		
Back lobe level (dB)	≤ − 20			≤ − 15		
Radiation pattern	Unidirectional			Unidirectional		
Mutual coupling (|*S*_12_| dB)	≤ − 40			≤ − 38		
Antenna efficiency, %	> 90			–		
*ECC*	≤ 0.002 (*ECC* ≤ 0.5 is acceptable)			≤ 0.001 (*ECC* ≤ 0.5 is acceptable)		
*DG* (dB)	≥ 9.99 (*DG* ≥ 9.95 is acceptable)			≥ 9.99 (*DG* ≥ 9.95 is acceptable)

**Table 3:** Comparison between the proposed antenna scheme and previous MIMO antennas for mobile terminals.

References	Frequency (GHz)	Gain (dBi)	Element size (mm)	Number of elements	Isolation (dB)	*ECC*	Devices
^11^	3.4–3.6	Not Given	15 × 15	8	10	0.2	Smartphone
^13^	3.4–3.6	Not Given	20.5 × 15	8	17.5	0.05	Smartphone
^20^	3.3–4.2	Not Given	52.9 × 45.8	3	15	0.1	Access point
^27^	3.4–3.6	Not Given	9.4 × 6.9	4	17	< 0.1	Mobile
^30^	2.5–7.0	Not Given	13.9 × 5.7	8	17	0.1	Mobile
^33^	2.7–3.6	3	16 × 33 × 3	2	25	< 0.009	Portable
^36^	3.4–3.6	Not Given	13.6 × 7	4	> 11 dB	0.195	Tablet
^38^	0.7–0.95 1.7–2.7	0.5–1.9 1.8–3.8	130 × 3	2	> 16 dB	< 0.2	Laptop
^39^	3.3–3.6 4.8–5.0	Not Given	30 × 0.6	4	> 10 dB	< 0.3	Laptop
^40^	3.3–3.6 4.8–5.0	Not Given	12.5 × 2 × 3	2	≥ 10	< 0.12	Laptop
Proposed antenna scheme	3.4–6.0	3.265–4.878	30.98 × 4.2	2	≥ 38 dB	< 0.001	Laptop

The envelope correlation coefficient (*ECC*) of the proposed broadband unidirectional twin-element MIMO antenna scheme is calculated based on the S-parameters (|*S*_11_|, |*S*_22_| and |*S*_12_|) using Eq. (1). The *ECC* of a MIMO antenna indicates the independence between the radiation patterns of the two single-element antennas. A high *ECC* indicates high correlation and low mutual coupling between the two antenna elements, resulting in poor antenna performance. On the other hand, if one antenna is completely horizontally polarized and the other is completely vertically polarized, the two antennas would have a correlation (i.e., *ECC*) of zero.

**Figure 26:**
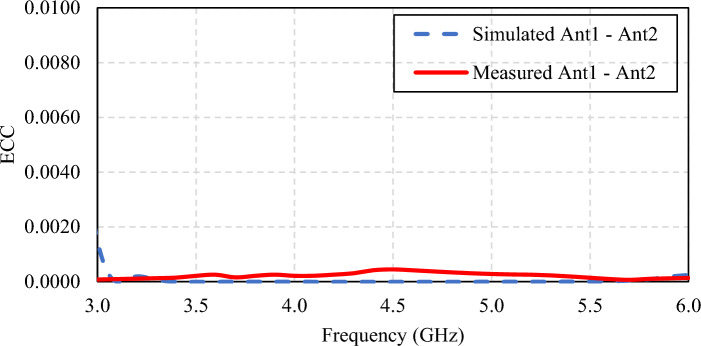
Simulated and measured *ECC* of the broadband unidirectional twin-element MIMO antenna scheme.

To measure *ECC* of the proposed MIMO antenna scheme, the reflection coefficient (|*S*_11_|) of Antenna element 1 and the transmission coefficient (|*S*_21_|) from Antenna element 1 to Antenna element 2 are first measured. The procedure is repeated for the reflection coefficient (|*S*_22_|) of Antenna element 2 and the transmission coefficient (|*S*_12_|) from Antenna element 2 to Antenna element 1. The *ECC* is calculated using Eq. 1, and Fig. 26 shows the simulated and measured *ECC* of the antenna scheme. The simulated and measured *ECC* of the proposed broadband unidirectional twin-element MIMO antenna scheme with the laptop model are less than 0.001 dB across the operating frequency band.

1 $$ ECC = \frac{{\left| {S_{11} * S_{12} + S_{22} * S_{21} } \right|^{2} }}{{(1 - \left| {S_{11} } \right|^{2} - \left| {S_{21} } \right|^{2} )(1 - \left| {S_{22} } \right|^{2} - \left| {S_{12} } \right|^{2} )}} $$

**Figure 27:**
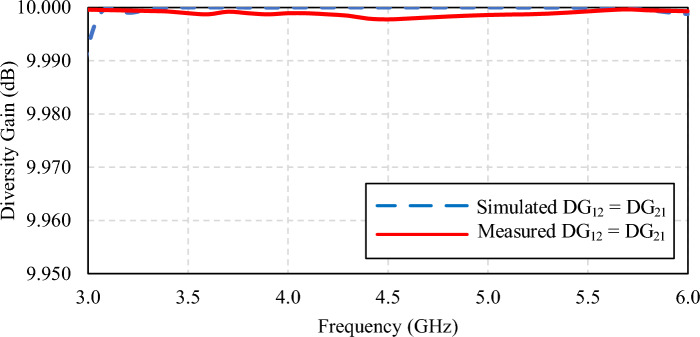
Simulated and measured diversity gain of the broadband unidirectional twin-element MIMO antenna scheme.

Diversity gain (*DG*) is a measure of reliability of the MIMO antenna scheme. A high *DG* indicates a high isolation between two single-element antennas of the MIMO antenna scheme. The *DG* of a MIMO antenna scheme is calculated by Eq. (2). Figure 27 shows the simulated and measured *DG* of the proposed broadband unidirectional twin-element MIMO antenna scheme with the laptop model. The simulated and measured *DG* are more than 9.990 dB.

2 $$ DG = 10\sqrt {1 - (ECC)^{2} } $$

## Conclusions

This research proposes the broadband unidirectional twin-element MIMO antenna scheme for mid-band 5G and WLAN applications (3.5–5.5 GHz). The twin-element antenna scheme comprises two single-element antennas on FR4 substrate. The single-element antenna consists of a T-shaped hemispherical feeding patch, left- and right-arm radiating patches, and a conjoined triangular ground plane. In the antenna design, the T-shaped hemispherical feeding patch is located between the left- and right-arm radiating patches separated by a coupling gap. The coupling gap is utilized to reduce the size of the antenna element while maximizing the impedance bandwidth. The proposed twin-element MIMO antenna scheme is integrated with the laptop model which functions as the reflector. The measured |*S*_11_| and |*S*_22_| are 55.32%, covering 3.4–6.0 GHz. The measured |*S*_12_| is less than − 15 dB, indicating no interference between the two single-element antennas. The measured gains of the proposed twin-element MIMO antenna scheme at 3.5, 4.5, and 5.5 GHz are 4.878, 4.585, and 3.265 dBi, respectively. The measured xz- and yz-plane cross-polarization levels are less than − 25 dB and − 15 dB. The HPBW in the xz-plane at 3.5, 4.5, and 5.5 GHz are 99°, 92.8°, and 84.2°, and the corresponding HPBW in the yz-plane are 102°, 78°, and 102°. The measured xz- and yz-plane back lobe levels are below − 15 dB across the entire operating frequency band. The radiation pattern of the proposed twin-element MIMO antenna scheme is of unidirectionality. The *ECC* and *DG* of the twin-element antenna scheme are < 0.001 and > 9.99 dB, respectively. The proposed broadband unidirectional twin-element MIMO antenna scheme is thus operationally suitable for mid-band 5G/WLAN communication systems.

## Data Availability

Data is available from the corresponding author on reasonable request.
